# Evaluation of thoracic vertebrae in healthy White New Zealand rabbit (*Oryctolagus Cuniculus*): Computed tomographic and morphometric study

**DOI:** 10.1002/vms3.847

**Published:** 2022-06-28

**Authors:** Sarang Soroori, Omid Zehtabvar, Banafsheh Shateri Amiri, Amir Rostami, Yasamin Vali

**Affiliations:** ^1^ Department of Surgery and Radiology Faculty of Veterinary Medicine University of Tehran Tehran Iran; ^2^ Department of Basic Sciences, Faculty of Veterinary Medicine University of Tehran Tehran Iran; ^3^ Department of Internal Medicine, Faculty of Veterinary Medicine University of Tehran Tehran Iran; ^4^ Department of Companion Animals and Horses University of Veterinary Medicine Vienna Vienna Austria

**Keywords:** computed tomography, morphometry, thoracic vertebrae, White New Zealand rabbit

## Abstract

**Background:**

Computed tomography currently has a prominent role in diagnosis and evaluation of vertebral column. On the other hand, a thorough knowledge about vertebral column property in normal state is prerequisite an accurate diagnosis of different abnormalities in this region.

**Objective:**

The purpose of this study was to present a complete and exact descriptive and morphometric evaluation of thoracic vertebrae in rabbits with computed tomography. In images which were constructed by CT, several structures and different parts of the thoracic vertebrae have been named.

**Methods:**

Ten healthy, mature, White New Zealand rabbits were evaluated. The morphologic and morphometric parameters of the thoracic vertebrae were studied. In this study, several parameters of thoracic vertebrae, such as vertebral body height, spinous process height, transverse process length, transverse process width, etc., were measured by computed tomography.

**Results:**

Some parameters, such as spinal canal height, spinal canal width, pedicle length, pedicle width, end plate width, and endplate height, had no significant difference through thoracic vertebrae but other parameters, such as vertebral body height, transverse process length, transverse process width, spinous process angle, transverse process angle, and vertebral body length, had a significant difference.

**Conclusions:**

In this study, a comprehensive anatomic atlas of CT anatomy of the thoracic vertebrae was produced for use by veterinary radiologists, clinicians, and surgeons. Finally, we must mention these two important points: (1) Many of the differences observed between rabbits and humans are based on the way the trunks of these two creatures are located on the ground and the differences in the way their bodies move. (2) In studies that are done by modelling humans on animals, it should be noted that the terms used in animal anatomy are different and the names are used using the principles of veterinary anatomy.

## Graphical abstract

Transverse computed tomography images of seven cervical, first, second, third, fourth, eleventh, and twelfth thoracic vertebrae.

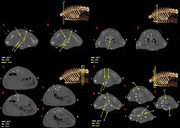

## 1 INTRODUCTION

Computed tomography (CT) is an extremely useful diagnostic technique for evaluating the brain, vertebral column, and all calcified structures in animals (Wilke et al., 1997).

As a reliable diagnosis and detection of abnormalities needs a sufficient knowledge of normal computed tomographic anatomic features, this study was conducted to present a comprehensive anatomic atlas of CT anatomy of the thoracic vertebrae in rabbits. CT is a non‐aggressive modality that provides detailed information about the vertebral column (ElRakhawy et al., 2010). Therefore, this modality is considered as a valuable method for the detection of bony changes in vertebrae in medicines and veterinary medicines (Axlund et al., 2003). However, there are some studies available presenting CT characteristics of normal dental structures (Da Costa et al., 2010) and teeth abnormalities (Da costa et al., 2010; ElRakhawy et al., 2010) as well as soft tissue abnormalities (da Costa et al., 2010). Despite the wide usage of rabbits in research, as yet, there is no study on the normal structure of different parts of the vertebral column by CT. Moreover, more detailed descriptions of structures of this region in normal position can be helpful for diagnosis of different diseases and abnormalities. It should be noted that studies on the morphometry of rabbit cervical vertebrae have been performed by CT scan (Shateri Amiri et al., 2020). Of course, cases, such as vertebral formula and congenital abnormalities of the vertebral column in rabbits, have also been considered in studies (Proks et al., 2018). Studies have also been performed on functional neuroanatomy of the domestic rabbit (Osofsky et al., 2007). Alfraihat et al. (2020) studied thoracic vertebral morphology in normal and scoliosis deformity in skeletally immature rabbits: In longitudinal study, they examined changes in the VBH (vertebral body height).

Comparative studies between rabbit vertebrae and other animals have also been performed, such as comparison of back and loin locomotor bony structures in cats and rabbits (thoracic and lumbar vertebrae) (Mohamed & El‐Hady, 2019). Of course, different parameters have been examined with our study, and there are similar cases that we compared with our study.

As noted, the usage of CT provides evaluation of organs in the body in their dynamic state and that may be practical. The purpose of this study is to present a complete and exact descriptive and morphometric evaluation of thoracic vertebrae in rabbits with CT.

## 2 MATERIAL AND METHODS

### 2.1 Animals

In this study, 10 female mature, White New Zealand rabbits (*Oryctolagus cuniculus*) with an average body weight of 1.95 ± 0.05 kg were evaluated, all of them were healthy physically. All the animals were selected randomly and were kept at the laboratory in the Faculty of Veterinary Medicine, University of Tehran, under the same standard living conditions such as caging and feeding. All the experimental procedures were approved by the University of Tehran based on the ethical values of the Institute for Laboratory Animal Research (2011) NIH *Guide for the Care and Use of Laboratory Animals*.

### 2.2 Computed tomography

The rabbits were scanned under general anaesthesia (Ketamine and Xylazine 0.3 mL/kg) (Carpenter et al., 2018) in ventral recumbency by using a helical scanner (Somatom Spirit Series II CT scanner, Siemens, Berlin, Germany) at the Small Animal Teaching Hospital of the Faculty of Veterinary Medicine, University of Tehran. At this stage, images were taken as transverse and perpendicular to the vertebral column and in 2 mm slices. Similar CT technical factors were used for all the scans (rotation time, 1 s; slice thickness, 1 mm; reconstruction interval, 0.5–1 mm; pitch, 1; X‐ray tube potential, 120 kV; and X‐ray tube current, 130 mA) (Silverman, 1993). The images were recorded and analysed subjectively by a national board‐certified veterinary radiologist. The scan of each patient was evaluated separately using Somaris/Syngo 5.5 software (2010, Siemens AG). Anatomical structures, such as spinous process, cranial articular process, vertebral body, transverse process, caudal articular process, ribs, and sternum, were detected and labelled in transverse images and 3D reconstruction. The window level and window width were adjusted manually based on the preference of the radiologist.

### 2.3 Morphometric study

For the objective part of the study, morphometric measurements were done by using the ruler tool of Somaris/Syngo 5.5 software (2010, Siemens AG). The measured parameters are described in Table 1. The measurements were recorded for each rabbit separately and they were analysed by SPSS 16.0 software and a paired sample *t* test (*p* > 0.05).

**TABLE 1:** Morphometric parameters of the of thoracic vertebrae of the rabbit

Parameters	Abbreviation	Description
Vertebral body height	VBH	Distance between the base of vertebra to vertebral canal in transverse view
Spinous process height	SPH	Distance between base of spinous process to apex of process in transverse view
Transverse process length	TPL	Distance between the base of transverse process to extremity of process in transverse view
Transverse process width	TPW	Distance between left extremity of process to right extremity in transverse view
Spinous process angle	SPA	The angle between spinous process with horizontal line in sagittal view
Transverse process angle	TPA	The angle between transverse process with horizontal line in transverse view
Spinal canal depth	SCD	Distance between proximal extremity of vertebral canal to distal extremity of vertebral canal in transverse view
Spinal canal width	SCW	Distance between left extremity of vertebral canal to right extremity of vertebral canal in transverse view
Pedicle length	PDL	Distance between proximal extremity of pedicle to distal extremity in transverse view
Pedicle width	PDW	The width of pedicle in transverse view
Vertebral body length	VBL	The length of vertebral body in sagittal view
Endplate width	EPW	The width of end plate in transverse view
Endplate height	EPH	The height of end plate in transverse view

## 3 RESULTS

### 3.1 Subjective evaluation

**FIGURE 1:**
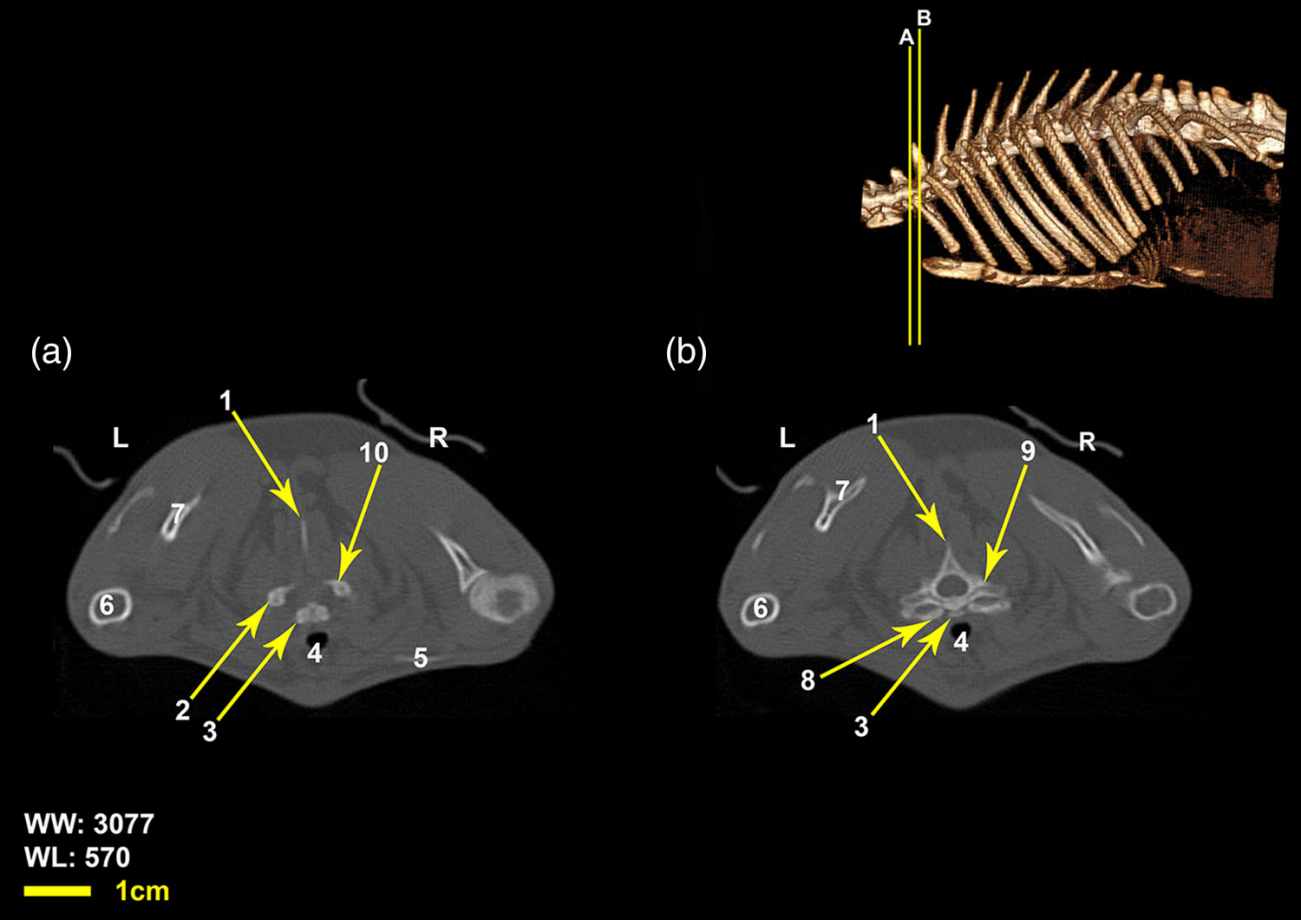
Transverse Computed tomography images (bone window) of the seventh cervical vertebrae and the first thoracic vertebrae in rabbit, the picture at the right top of this figure shows the section of transverse CT images (3D reconstruction image, Osseous‐Shaded‐vp). (1) Spinous process of the first thoracic vertebra, (2) cranial articular process of the first thoracic vertebra, (3) body of the seventh cervical vertebra, (4) trachea, (5) clavicle, (6) humerus, (7) scapula, (8) the first rib, (9) transverse process of the first thoracic vertebra, (10) caudal articular process of the seventh vertebra

**FIGURE 2:**
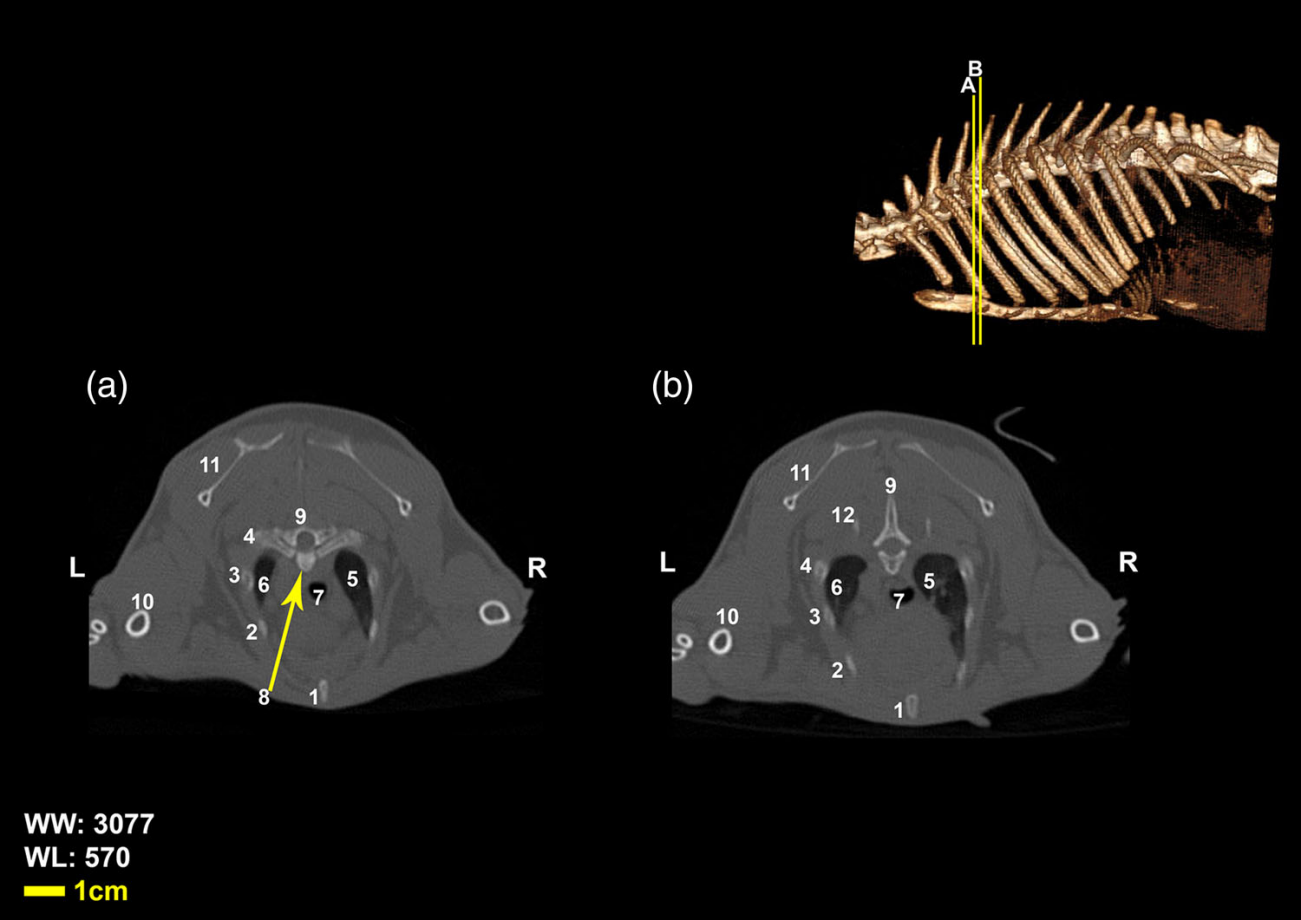
Transverse Computed tomography images (bone window) of the first, second, and third thoracic vertebrae in rabbit, the picture at the right top of this figure shows the section of transverse CT images (3D reconstruction image, Osseous‐Shaded‐vp). (1) Body of the first thoracic vertebra, (2) first rib, (3) caudal articular process of the first thoracic vertebra, (4) trachea, (5) cranial articular process of the second vertebra, (6) humerus, (7) scapula, (8) body of the second thoracic vertebra, (9) sternum, (10) spinous process of the second thoracic vertebra, (11) transverse process of the second thoracic vertebra, (12) the second rib, (13) third rib, (14) the third thoracic vertebra, (15) the fourth rib, (16) right lung, (17) spinous process of the third thoracic vertebra

**FIGURE 3:**
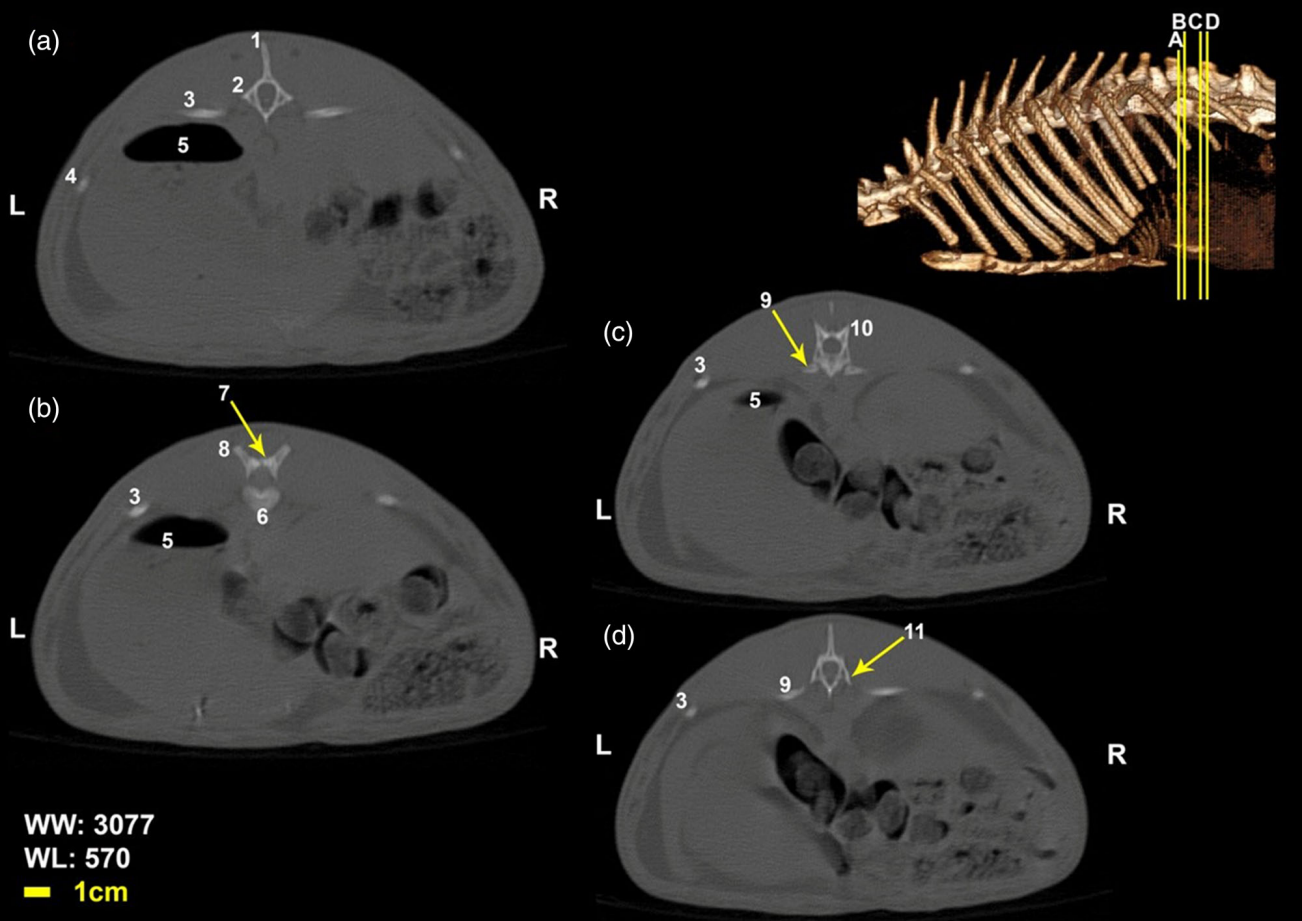
Transverse Computed tomography images (bone window) of the third and fourth thoracic vertebrae in rabbit, the picture at the right top of this figure shows the section of transverse CT images (3D reconstruction image, Osseous‐Shaded‐vp). (1) sternum, (2) second rib, (3) third rib, (4) fourth rib, (5) right lung, (6) left lung, (7) trachea, (8) body of third thoracic vertebra, (9) fourth thoracic vertebra, (10) humerus, (11) scapula, (12) fifth rib

**FIGURE 4:**
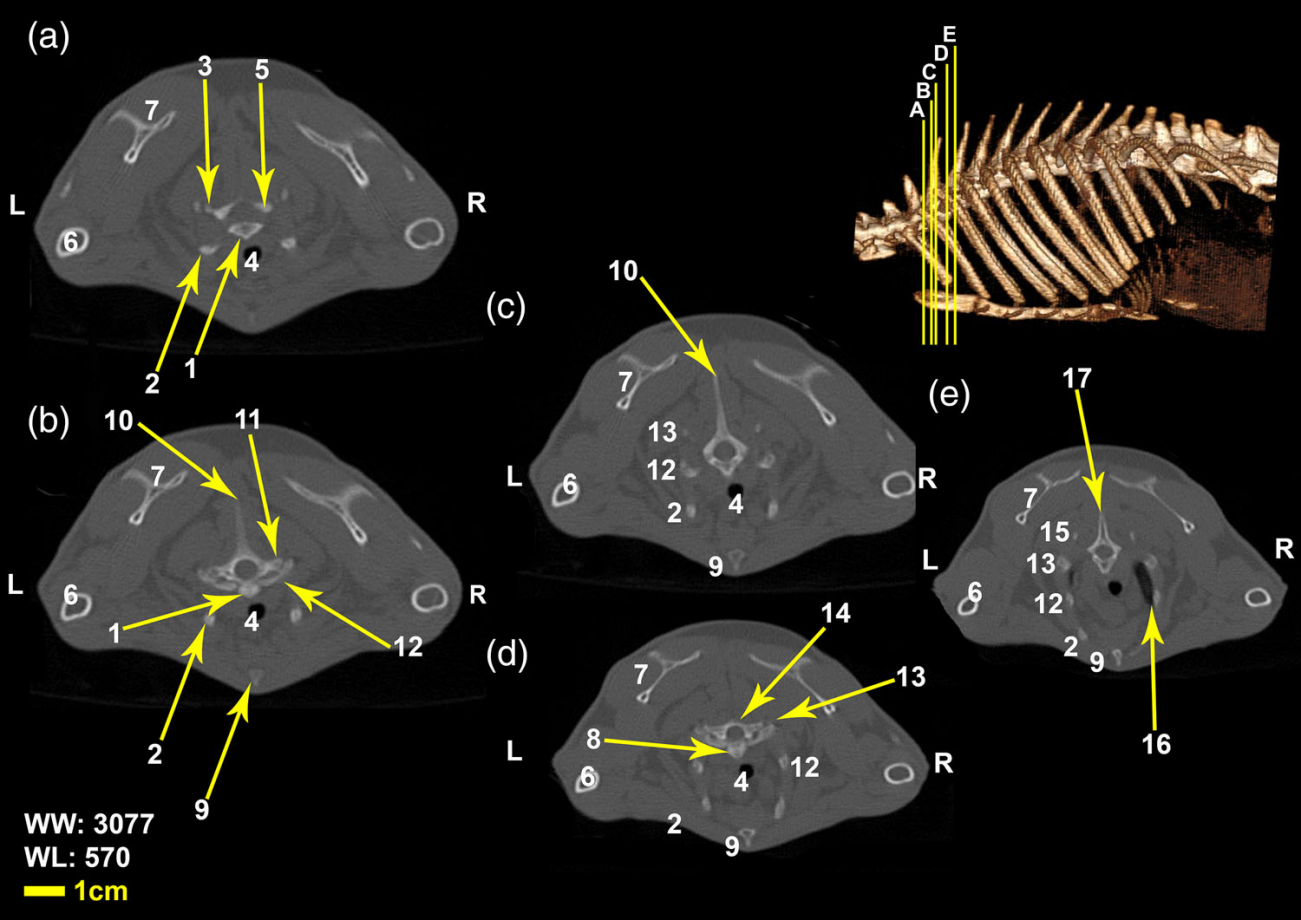
Transverse Computed tomography images (bone window) of the 11th and 12th thoracic vertebrae in rabbit, the picture at the right top of this figure shows the section of transverse CT images (3D reconstruction image, Osseous‐Shaded‐vp). (1) Spinous process of 11th vertebra, (2) transverse process of 11th vertebra, (3) the 11th rib, (4) the 10th rib, (5) stomach, (6) body of 11th vertebra, (7) caudal articular process of 11th vertebra, (8) cranial articular process of the 12th vertebra, (9) the 12th rib, (10) the 12th thoracic vertebra, (11) transverse process of the 12th vertebra

**FIGURE 5:**
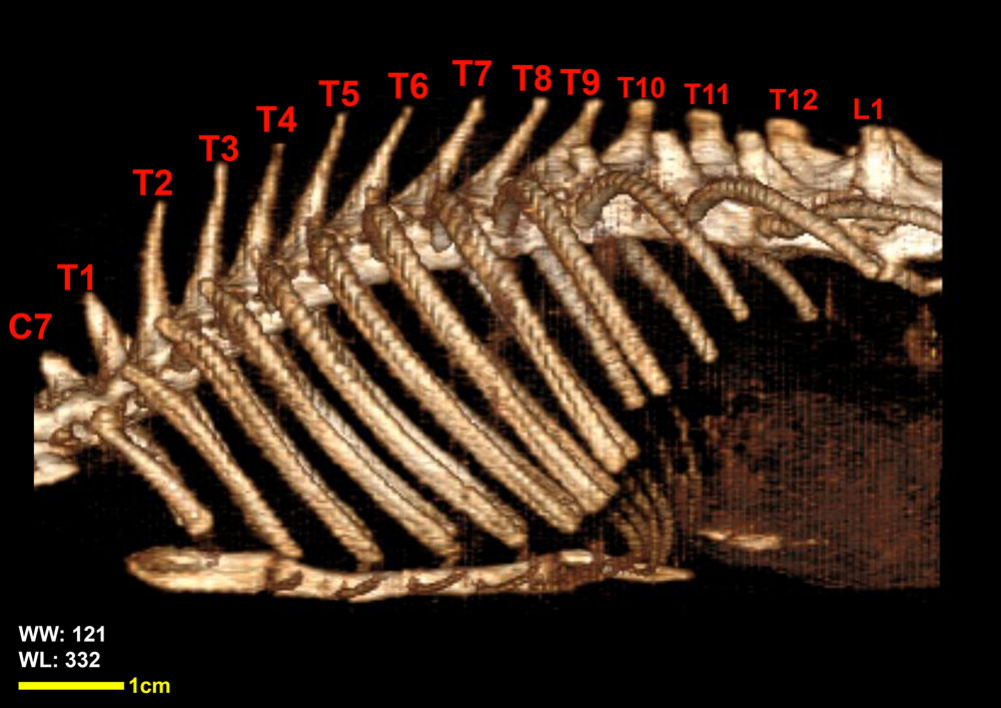
3D reconstruction image, Osseous‐Shaded‐vp, thoracic vertebrae in rabbit

Twelve vertebrae existed in the thoracic region of all the included White New Zealand rabbits. Bodies of thoracic vertebrae were shorter than cervical vertebrae and spinous processes were directed caudally in cranial thoracic vertebrae (Figure 1). A vertical spinous process, that is called an anticlinal vertebra, was detected in the tenth thoracic vertebra. From this region, spinous processes were directed cranially. Articular process in these vertebrae was small and was present in two pairs, one pair of cranial articular processes was located dorsally and the other ventrally (Figures 2, 3, 4). Transverse process in these vertebrae was small and had an articular surface for articulation with rib prominence (Figures 1, 2, 4, and 5). Spinous process in these vertebrae was long (Figures 1, 2, 3, 4). Accessory processes were in the 9th to 12th thoracic vertebrae. Mammillary processes were in the 10th to 12th thoracic vertebrae.

### 3.2 Objective evaluation

The results of mensuration of the mentioned parameters have been analysed by SPSS 16.0 software and a paired sample *t* test (*p* > 0.05).

VBH had an invariable measure in thoracic vertebrae (*p* > 0.05). The difference of SPH was statistically significant between the second vertebrate and other thoracic vertebrae and also between the third thoracic vertebrae and other thoracic vertebrae (*p *< 0.05). However, the SPH did not reach the significant level between the second and third vertebrae. The 12th vertebrae showed a statistically different TPL and TPW in comparison to lumbar and the other thoracic vertebrae, respectively (*p* < 0.05). The mean of SPA was statistically different between the first and second thoracic vertebrae (*p* < 0.05). SPA of the tenth thoracic vertebrae was measured almost at 90° and in White New Zealandian it is considered as an anticlinal vertebra. The difference in this parameter was statistically significant between the third to ninth thoracic vertebrae, and the first to second vertebrae (*p* < 0.05). The difference of this parameter was statistically significant between the tenth and the third to the tenth and also with the first and second thoracic vertebrae (*p* < 0.05). The difference of this parameter is statistically meaningful between 11th and 12th thoracic vertebrae in comparison to tenth, between the third to ninth vertebrae in comparison to the first and second vertebrae (*p *< 0.05). The difference of TPA was statistically valid between the ninth to 11th thoracic vertebrae in comparison to the second to eighth vertebrae (*p* < 0.05) and also between the first thoracic vertebra in comparison to the second to eighth thoracic vertebrae (*p* < 0.05). The difference VBL was statistically significant between the 12th thoracic vertebra and the rest of the thoracic vertebrae (*p* < 0.05). The difference of PDW, EPH, EPW, PDL, SCW, and VBH did not reach a statistical level between thoracic vertebrae (*p* > 0.05). The results of measurements and statistical analysis are shown in Tables 2 and 3.

**TABLE 2:** Computed tomographic measurements of thoracic vertebrae of the rabbit, mean ± SD (standard deviation), in cm and degree*

Thoracic vertebrae	TPA^*^	SPA^*^	TPW	TPL	SPH	VBH
**T_1_**	10.2 ± 0.6a	66.1 ± 5.9a	1.5 ± 0.2a	0.7 ± 0.1a	1.2 ± 0.2a	0.4 ± 0.06a
**T_2_**	18.4 ± 0.3b	46.2 ± 5.9b	1.5 ± 0.07a	0.6 ± 0.04a	1.6 ± 0.1b	0.4 ± 0.03a
**T_3_**	20.7 ± 0.1b	35.9 ± 0.2c	1.6 ± 0.06a	0.6 ± 0.03a	1.7 ± 0.2b	0.5 ± 0.04a
**T_4_**	19.8 ± 1.05b	34 ± 0.5c	1.4 ± 0.09a	0.6 ± 0.06a	1.1 ± 0.1a	0.4 ± 0.04a
**T_5_**	19 ± 0.9b	33.7 ± 0.9c	1.4 ± 0.07a	0.6 ± 0.04a	1.2 ± 0.1a	0.4 ± 0.03a
**T_6_**	21.2 ± 0.1b	32 ± 0.3c	1.4 ± 0.04a	0.5 ± 0.04a	1.2 ± 0.1a	0.4 ± 0.03a
**T_7_**	18.8 ± 0.4b	31.6 ± 0.8c	1.4 ± 0.09a	0.5 ± 0.03a	1.1 ± 0.1a	0.4 ± 0.03a
**T_8_**	19.3 ± 0.1b	33.4 ± 0.4c	1.3 ± 0.1a	0.5 ± 0.06a	1 ± 0.1a	0.4 ± 0.03a
**T_9_**	12.8 ± 0.3c	34.6 ± 3.2c	1.4 ± 0.2a	0.5 ± 0.1a	0.9 ± 0.04a	0.5 ± 0.04a
**T_10_**	13.4 ± 6c	89.5 ± 0.5d	1.5 ± 0.2a	0.6 ± 0.1a	0.9 ± 0.05a	0.5 ± 0.05a
**T_11_**	13.2 ± 0.4c	99.9 ± 0.9e	1.4 ± 0.1a	0.5 ± 0.06a	0.8 ± 0.06a	0.5 ± 0.05a
**T_12_**	43.3 ± 0.1d	100.6 ± 0.2e	0 ± 8.09b	0.1 ± 0.04b	0.8 ± 0.06a	0.5 ± 0.05a

The different letters (a,b,c,d,e) in each column represent significant difference between vertebrae (n = 10, *p* < 0/0.5).

**TABLE 3:** Computed tomographic measurements of thoracic vertebrae of the rabbit, mean ± SD (standard deviation), in cm and degree^*^

Thoracic vertebrae	EPH	EPW	VBL	PDW	PDL	SCW
**T_1_**	0.4 ± 0.05a	0.7 ± 0.07a	0.7 ± 0.08a	0.3 ± 0.03a	0.4 ± 0.08a	0.6 ± 0.04a
**T_2_**	0.4 ± 0.05a	0.6 ± 0.08a	0.7 ± 0.09a	0.2 ± 0.04a	0.4 ± 0.05a	0.5 ± 0.02a
**T_3_**	0.4 ± 0.02a	0.6 ± 0.08a	0.7 ± 0.06a	0.2 ± 0.04a	0.4 ± 0.07a	0.5 ± 0.02a
**T_4_**	0.4 ± 0.02a	0.5 ± 0.09a	0.7 ± 0.09a	0.2 ± 0.03a	0.4 ± 0.06a	0.4 ± 0.03a
**T_5_**	0.3 ± 0.01a	0.6 ± 0.1a	0.7 ± 0.1a	0.2 ± 0.02a	0.4 ± 0.05a	0.4 ± 0.04a
**T_6_**	0.3 ± 0.01a	0.6 ± 0.1a	0.7 ± 0.08a	0.2 ± 0.03a	0.4 ± 0.06a	0.4 ± 0.04a
**T_7_**	0.3 ± 0.06a	0.6 ± 0.1a	0.7 ± 0.05a	0.2 ± 0.03a	0.4 ± 0.08a	0.4 ± 0.05a
**T_8_**	0.4 ± 0.03a	0.7 ± 0.1a	0.8 ± 0.09a	0.2 ± 0.03a	0.4 ± 0.07a	0.4 ± 0.05a
**T_9_**	0.4 ± 0.05a	0.7 ± 0.07a	0.9 ± 0.04a	0.2 ± 0.03a	0.3 ± 0.07a	0.4 ± 0.03a
**T_10_**	0.4 ± 0.04a	0.7 ± 0.06a	1 ± 0.08a	0.3 ± 0.08a	0.3 ± 0.07a	0.4 ± 0.03a
**T_11_**	0.4 ± 0.05a	0.8 ± 0.04a	0.9 ± 0.05a	0.2 ± 0.04a	0.4 ± 0.04a	0.4 ± 0.04a
**T_12_**	0.4 ± 0.02a	0.8 ± 0.04a	1.5 ± 0.07b	0.2 ± 0.03a	0.4 ± 0.07a	0.4 ± 0.03a

The different letters (a,b) in each column represent significant different between vertebrae (n = 10, *p* < 0/0.5).

## 4 DISCUSSION

The present study is a descriptive design to evaluate the computed tomographic features of the thoracic vertebrae in rabbits. This study described the specific morphometric criteria of the thoracic vertebra. However, CT scan was introduced as one of the most practical diagnostic methods for small animal orthopaedics purposes (Axlund et al., 2003). The computed tomographic anatomy of neck, thorax, and abdomen in healthy rabbits was first described by Zotti et al. (2009). Later, (Da Costa et al. 2010) presented CT as a fast and exact method for evaluation of vertebral column in small animals and computed tomographic features of intervertebral degenerative diseases in rabbits. Wilke et al. (1997) evaluated anatomy of vertebral canal in sheep and compared it to human anatomy. In the latter study, five cases were evaluated and same objective parameters were measured. Five complete spines were measured to determine 21 dimensions from the pedicles, spinal canal, transverse, and spinous processes, facets, endplates, and disc. The results showed that sheep and human vertebrae are most similar in the thoracic and lumbar regions, although they show substantial differences in certain dimensions. Egwu et al. (2019) evaluated VBH parameters on the human body. Of course, it should be noted that the parameter measured as VBL in rabbits is equivalent to parameter VBHm in humans due to the position of the animal's body on the ground. Parameter VBL in the rabbits, as mentioned, does not change, although it increases in the last thoracic vertebra. The parameter measured as VBH in rabbits is equivalent to parameter VBLm in humans. Based on a study by Egwu et al. (2019) in humans, the VBLm had a minimum value at T2. There was a gradual increase from T2 to the maximum at T9. Having extensively searched through numerous engines, according to our study in rabbits, the size of VBH was constant and did not change. Alfraihat et al. (2020) studied changes in the VBH in the thoracic vertebrae in normal and scoliosis deformity in skeletally immature. We should mention that the parameter that they have examined as VBH is based on the naming of human anatomy, and in fact, based on the way the rabbit's body is placed on the ground, the term VBL should be used for this parameter, which is also used in our study (Alfraihat et al., 2020).

Furthermore, Tan et al. (2004) showed that the TPL parameter on the human body has the maximum length at L3, however, in the current study, the 12th vertebrae showed a statistically different TPL in comparison to the lumbar and the other thoracic vertebrae. In this study, the TPW parameter has been also evaluated and showed a significant reduction from T1 to T5 and then became constant up to T8 and then again decreased from T8 to T12, however, in the current study, the 12th vertebrae showed a statistically different TPW in comparison to lumbar and the other thoracic vertebrae. Additionally, they also presented that SCW parameter was constant from C3 to C5 and then had 5 mm increased in width between T1 and T4 and again this parameter was measured constant up to T10 and then showed an increase in width up to T12. In lumbar vertebrae, SCW parameter was constant from L1 to L3 and then at the location of L5 had the maximum width, which in current study SCW did not reach a statistical level between thoracic vertebrae (Tan et al., 2004).

Kaur et al. (2016) evaluated TPL parameters and revealed that the length of the transverse process increases from T1 to T7 and then decreases at the location of T12. They also revealed that in thoracic vertebrae, T4 to T5 have the least SCW (Kaur et al., 2016). Elrakhawy et al. (2010) evaluated human lumbar vertebrae and presented a gradual increase of SCW parameters except at L3. CT is increasingly used in exotic pet medicines due to public awareness, higher standards of care demand and the increased availability in small animal practice (Kaur et al., 2016). Orthopaedic diseases are a common problem in exotic pets. Diagnostic imaging modalities are more accessible and available in exotic animal veterinary medicine, the higher standards of care of the veterinary profession have progressed toward an increased offer of advanced imaging modalities (Kaur et al., 2016). In some diseases, such as metabolic bone disease, neoplasia, degenerative intervertebral disc disease, and compression fractures, the size and the shape of the vertebrae change and compression with the normal shape and size could be diagnostic. In rabbits, the most common reason of acute posterior paralysis is vertebral fracture or luxation (Kaur et al., 2016). In this study, computed tomographic anatomy of thoracic vertebrae in 10 White New Zealand rabbits was evaluated and different parts of vertebrae have been named and described and also almost all parameters of vertebra in thoracic region were measured in CT.

CT is an extremely useful diagnostic technique for evaluating the brain, vertebral column, and all calcified structures in rabbits (Wilke et al., 1997). The purpose of this study was to produce a comprehensive anatomic atlas of CT anatomy of the thoracic vertebrae for the use by veterinary radiologists, clinicians, and surgeons. In conclusion, VBH had an invariable measure in thoracic vertebrae and also it had an invariable measure through the whole lumbar vertebrae. SPH increased at the second thoracic vertebra to the location of the fourth thoracic vertebra, and then it decreased and was invariable up to the location of the seventh lumbar vertebra. TPL was invariable up to the 11th thoracic vertebra. It decreased at the 12th thoracic vertebra and then again it increased at the location of the first lumbar vertebra. Finally, we must mention these two important points: (1) Many of the differences observed between rabbits and humans are based on the way the trunks of these two creatures are located on the ground and the differences in the way their bodies move. (2) In studies that are done by modelling humans on animals, it should be noted that the terms used in animal anatomy are different and the names are used using the principles of veterinary anatomy.

## CONFLICT OF INTEREST

The authors declare that they have no conflict of interest.

## ETHICAL DISCLOSURE

The authors declare that the study was conducted according to the national guidelines without causing harm to the animals and respecting their welfare.

### PEER REVIEW

The peer review history for this article is available at https://publons.com/publon/10.1002/vms3.847.

## Data Availability

No data are available.
